# Idiopathic Sudden Sensorineural Hearing Loss, But Not Compatible With the Classical Definition

**DOI:** 10.7759/cureus.47472

**Published:** 2023-10-22

**Authors:** Bahar Kayahan Sirkeci

**Affiliations:** 1 Otolaryngology - Head and Neck Surgery, Liv Hospital, Ankara, TUR

**Keywords:** acute inner ear dysfunction, prognostic factors, atypical audiogram pattern, hearing impairment, sudden sensorineural hearing loss

## Abstract

Background and purpose: The purpose of this study was to raise awareness for patients who did not meet the audiometric criteria of idiopathic sudden sensorineural hearing loss (SSNHL) but complained of acute hearing loss.

Materials and methods: Medical records of patients who were diagnosed with SSNHL from October 2021 to March 2023 were examined retrospectively. Among 223 patients with SSNHL, 40 cases with atypical SSNHL and fitting in the criteria were included in the study. The patients who were included in this study were the ones who were given one bolus dose of IV systemic steroid (250 mg methylprednisolone with a proton pump inhibitor) and betahistine 2x24 mg po for a month. Pure tone audiometry was performed during the initial visit, on the fifth day, and at the end of the one-month usage of betahistine tablets. Hearing levels on the 250, 500, 1,000, 2,000, 4,000, and 8,000 Hz of the affected ear were compared with those of the contralateral ear. Hearing improvement was calculated as the hearing gain (in decibels) on the control audiograms and the resolution of the patients' complaints.

Results: These patients were suffering from idiopathic SSNHL with minimal hearing impairment. A total of 36 of the cases had hearing recovery on the fifth-day audiogram, and the remaining four patients showed hearing improvement on the first-month audiogram. The changes between the initial and the control audiogram values were found to be statistically significant (p<0.001). Additionally, these patients showed distinctive characteristics, such as being younger than the classical SSNHL population, lacking systemic diseases (diabetes or hypertension), and having good unaffected hearing.

Conclusion: Although there have been lots of studies to understand the pathophysiology, prognostic factors, and treatment options for SSNHL, atypical SSNHL patients have been underestimated and generally excluded from studies, and there have been a small number of studies on this issue. These patients might be accepted as having mild hearing loss. However, when the nerve injury is proven by audiograms, it is hard to decide what to do due to the lack of a treatment approach. This study is important as it focuses on atypical SSNHL cases. Further studies with larger group patients or prospective randomized-controlled group studies are needed to define these patients and decide how to treat this type of acute nerve dysfunction.

## Introduction

Sudden sensorineural hearing loss (SSNHL) is defined as a hearing impairment in one or both ears with an early onset within three days, in the sensorineural pattern of at least 30 dB over three consecutive frequencies [1,2]. SSNHL with no organic pathology through investigation is defined as idiopathic SSNHL [1-3]. The most common theories include viral infection, rupture of the cochlear membrane, and vascular etiologies. The treatment options appear still controversial, and different therapeutic options have been questioned and recommended to cure acute inner ear dysfunction [1,4]. Nevertheless, there have been many cases that have atypical audiograms with a history of SSNHL, less than 30 dB HL compared to the other ear, or have HL only in one or two consecutive frequencies, or have both conditions. The treatment options for these patients are more controversial, having acute onset of complaints, but the audiograms do not fit in the classical knowledge [3,5]. In the literature, there have been some studies concerning these patients but not enough to determine an acceptable treatment schedule. This research aimed to understand the efficacy of one bolus dose of IV systemic steroid and betahistine 2x24 mg po for a one-month regimen on patients with atypical SSNHL.

## Materials and methods

Medical records of patients who were diagnosed with SSNHL from October 2021 to March 2023 were examined retrospectively. All patients had undergone audiological examination and magnetic resonance imaging (MRI) of the internal auditory canal to exclude any organic pathology. The age, sex, presence of systematic diseases, presence of tinnitus, ear fullness, sound sensitivity and/or dizziness, and the interval between the onset of symptoms and treatment were documented. The criteria for exclusion were patients who are under 18 years; have systematic diseases such as autoimmune diseases, diabetes, hypertension, and liver and kidney dysfunctions; have a previous history or recurrence of sensorineural hearing loss or Meniere disease; have a previous history of tinnitus; and who could not be observed for one month.

Among 223 patients with SSNHL, 40 cases with atypical SSNHL and fitting in the criteria were included in the study. The Institutional Review Board of Liv Hospital, Ankara, Turkey, approved this study (2023/010/010).

All patients who were included in this study were given one bolus dose of IV systemic steroid (250 mg methylprednisolone with a proton pump inhibitor) and betahistine 2x24 mg po for a month. Moreover, they were advised to avoid salt and caffeine, as routine advice for all SSNHL patients.

Pure tone audiometry was performed during the initial visit, on the fifth day, and at the end of the one-month usage of the betahistine tablet. Hearing levels on the 250, 500, 1,000, 2,000, 4,000, and 8,000 Hz of the affected ear were compared with those of the contralateral ear. Hearing improvement was calculated as the hearing gain (in decibels) compared to the initial audiograms and the resolution of the patients’ complaints.

Statistical analysis was performed by an expert statistician on the Excel file of patients' data by using Statistical Product and Service Solutions (SPSS) (version 20.0; IBM SPSS Statistics for Windows, Armonk, NY). The normality of the numerical data was examined with the Shapiro-Wilk test. Numerical data with normal distribution were analyzed using student-t and ANOVA tests, analysis of data not showing normal distribution was made using the Mann-Whitney U and Kruskal-Wallis tests, and repeated measurements were compared using Friedman and Wilcoxon tests. Pierson's chi-square test was used in the analysis of categorical data. The statistical significance level was accepted as p<0.05.

## Results

A total of 223 patients who complained of SSNHL were admitted to the clinic during the study period; 50 of the cases were diagnosed as atypical SSNHL, and 40 of them were included in the study as they underwent MRI examination. They were also given one bolus dose of IV systemic steroid (250 mg methylprednisolone with a proton pump inhibitor) and betahistine 2x24 mg po for a month. Thirty-eight of the patients had unilateral atypical hearing loss, and two had bilateral complaints, which were supported by audiological examination. There were 18 female (45%) and 22 male (55%) patients, and the mean age was 31.5±9.2 (ranging between 18 and 52 years). Half of the patients had atypical SSNHL on the left ear and 18 of them on the right ear (45%), and two cases had a bilateral loss (5%). The most common complaint was ear fullness (50%), 18 patients had sudden onset of tinnitus (45%), and two patients had hypersensitivity to sound (5%). Seventeen patients had ear complaints following viral infection (42.5%), 11 patients (27.5%) following acoustic trauma (loud noise/music, gunshot, etc.), eight patients with a history of stress (20%), and four cases did not have any prominent factor who was accepted as an unknown risk factor (10%). A brief summary of patients’ demographics and anamnesis of the SSNHL was demonstrated in Table 1. According to the results of the MRI examination, 35 (87.5%) of patients had normal inner ear configuration, whereas four of them had high juguler bulbus (10%), and one patient had a prominent anterior inferior cerebellar artery (AICA) loop. Pure tone audiometry, which was performed during the initial visit, showed that 14 of the cases had cochlear sensitivity (35%).

**Table 1:** The brief summary of patients' demographics and anamnesis of SSNHL.

		Number of patients (n/%)	Time elapsed until the start of treatment (day ±)	Mean Age (±sd)	Age Min.-Max.
Total		40 (%100)	2.2 (1.2)	31.5 (9.2)	18-53
Sex	Female	18 (%45)	2.4 (1.1)	30.3 (8.7)	19-48
	Male	22 (%55)	2.0 (1.2)	32.5 (9.7)	18-53
Main Complaint of Administration	Tinnitus	18 (%45)	1.9 (1.1)	29.5 (7.0)	21-41
	Ear Fullness	20 (%50)	2.5 (1.2)	34.1 (10.6)	18-53
	Sound Sensitivity	2 (%5)	2.0 (1.4)	23.5 (2.1)	22-25
Possible Etiology of Hearing Loss	Viral	17 (%42.5)	2.0 (1.2)	28.1 (10.1)	18-53
	Acoustic Trauma	11 (%27.5)	2.5 (1.4)	37.5 (5.8)	28-48
	Stress	8 (%20)	2.5 (0.9)	29.5 (5.5)	24-38
	Unknown Etiology	4 (%10)	1.8 (1.0)	33.5 (7.6)	23-41
Effected Side	Left	20 (%50)	2.4 (1.1)	30.6 (9.6)	19-53
	Right	18 (%45)	2.1 (1.3)	31.9 (9.3)	18-48
	Bilateral	2 (%5)	1.5 (0.7)	36.5 (2.1)	35-38

Follow-up appointments and audiological assessments were done on the fifth day of the treatment and at the end of the one-month usage of betahistine tablets. Hearing levels on the 250, 500, 1,000, 2,000, 4,000, and 8,000 Hz of the affected ear were compared with those of the contralateral ear. Only two cases had bilateral involvement, and it was found that the median value of the hearing loss was 10 dB (min: 5, max: 20 dB) compared to that of the collateral ear.

**Figure 1:**
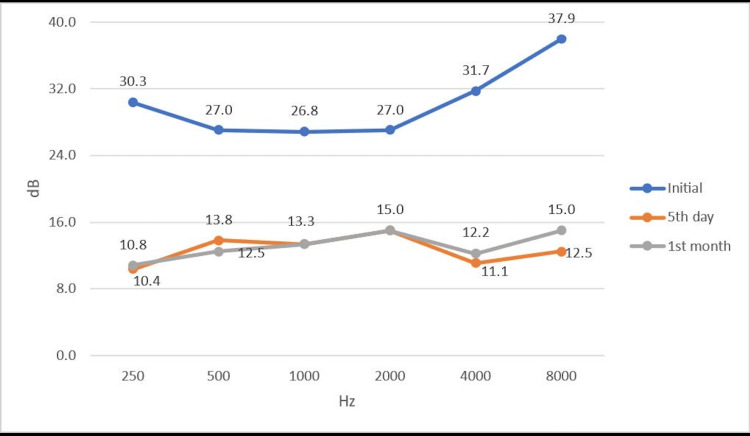
The median hearing improvement of the affected ear on the initial, fifth-day, and first-month control audiograms.

**Figure 2:**
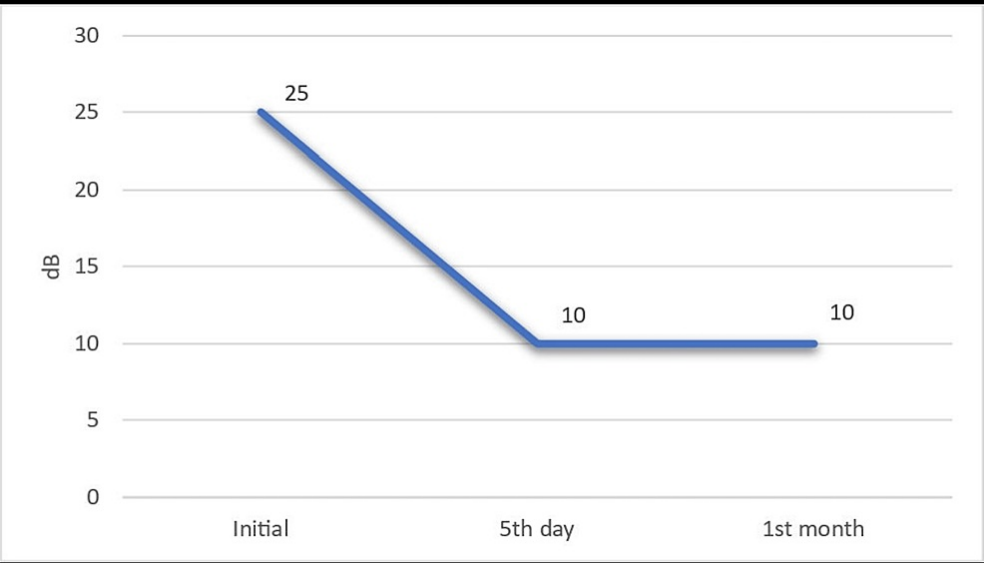
The audiological changes of the affected ear on the initial assessment and fifth day and first month tests.

The statistical analysis of the unilateral atypical hearing loss compared to that of the other ear was found to be statistically significant (p<0.001). The patients with bilateral involvement had 20 dB sensorineural hearing loss, one at 500-1,000 Hz and the other one at 1,000 Hz. These two cases were excluded in the case of this statistical analysis since the number of patients is quite low. The audiological changes of the affected ear on the initial assessment and fifth-day and first-month tests can be seen in Figures 1-2. The changes between the initial and control audiogram values were found to be statistically significant (p<0.001). Pairwise comparisons showed that all of these statistically significant differences were between the on-admission and fifth-day measurements. In addition, the differences between the fifth-day measurement values ​​and the first-month measurement values ​​were not found statistically significant.

When the initial complaints were distributed according to the possible etiology of anamnesis, it was seen that 46.7% of tinnitus was stress-related; additionally, 52.6% of the ear fullness had a recent viral infection history, and 42.1% of them had acoustic trauma. The differences between the factors causing the complaints of tinnitus and fullness were found as statistically significant (p=0.018) (Table 2).

**Table 2:** Evaluation of the main complaints and the possible cause of hearing loss. The differences between the factors causing the complaints of tinnitus and ear fullness were found as statistically significant (p=0.018). Stress was found to be more likely to cause tinnitus, whereas viral etiology was more prominent for ear fullness complaints (*The patients with unknown risk factors were excluded, as well as the patients with sound sensitivity since the number of patients is quite low).

			Possible etiology			Total
			Viral	Acoustic trauma	Stress	
Main complaint	Tinnitus	n	5	3	7	15
		%	33.3%	20.0%	46.7%	100.0%
	Ear fullness	n	10	8	1	19
		%	52.6%	42.1%	5.3%	100.0%
Total		n	15	11	8	34
		%	44.1%	32.4%	23.5%	100.0%

The possible etiology (acoustic trauma, viral infection, or stress) did not have any statistical significance on the side of the affected ear (Table 3). Further, there was no statistically significant difference when the cochlear sensitivity was compared with the possible etiology of atypical SSNHL (Table 4).

**Table 3:** Affected ear side and possible cause of hearing loss. The possible etiology (acoustic trauma, viral infection, or stress) did not have any statistical significance on the side of the affected ear (p=0.166). *The patients with unknown risk factors were excluded, as well as the patients with bilateral hearing loss since the number of patients is quite low.

			The possible cause			Total
			Viral	Acoustic trauma	Stress	
Affected ear side	Left	N	9	3	5	17
		%	52.9%	17.6%	29.4%	100.0%
	Right	N	8	8	2	18
		%	44.4%	44.4%	11.1%	100.0%
Total		N	17	11	7	35
		%	48:6%	31.4%	20.0%	100.0%

**Table 4:** Cochlear sensitivity existence on the audiogram and possible etiology of hearing loss. *The patients with unknown risk factors were excluded since the number of patients is quite low. There was no statistically significant difference observed between cochlear sensitivity and the possible etiology of atypical SSNHL (p=0.324).

			Possible etiology			Total
			Viral	Acoustic trauma	Stress	
Cochlear sensitivity	No	n	13	6	4	23
		%	56.5%	26.1%	17.4%	100.0%
	Yes	n	4	5	4	13
		%	30.8%	38.5%	30.8%	100.0%
Total		n	17	11	8	36
		%	47.2%	30.6%	22.2%	100.0%

## Discussion

SSNHL, which is also known as sudden deafness, is defined as a sudden onset of hearing loss frequently without a detectable pathology. SSNHL, a disease of unknown etiology in about 90% of cases, is defined as more than 30 dB sensorineural hearing loss occurring at least three consecutive frequencies within 72 hours or less [1-4]. It is one of the otolaryngological emergencies, and its incidence is not so uncommon. The incidence of SSNHL is estimated to be approximately 5-30 per 100,000, and it has a tendency to occur between 40 and 65 years of age, and gender distribution is equal [2,6,7]. Although the pathophysiology of SSNHL is still unclear, many factors, including infection, microvascular occlusion, auto-immune conditions, and coagulation disorders, are suggested to play a role in the etiology. The treatment protocols and even the necessity of the treatment were still controversial, and spontaneous recovery rates of up to 65% were reported, which suggested a good prognosis [8,9]. Moreover, there have been quite a number of research putting forward that, if SSNHL was not given immediate treatment, it might cause serious disorders, such as permanent hearing impairment, tinnitus, and psychiatric sequelae. Poor prognostic factors were described as advanced age, the presence of vertigo, the severity of initial hearing loss, the time interval between the onset of symptoms and treatment, and the presence of comorbid diseases, such as diabetes mellitus or hypertension [10,11]. Since the etiology and pathogenesis of SSNHL are still uncertain, there is not any standard treatment protocol. The most commonly used treatment option for SSNHL has been corticosteroid therapy, which could be administered as oral, intravenous, or intratympanic injections. In most cases, multiple therapeutics were given at the same time as soon as possible because the exact cause could not be detected and the immediate treatment onset has been still valuable. Steroids generally might be used in combination with one or more agents, such as betahistine, acyclovir, dextran, ginkgo biloba, nifedipine, magnesium, vitamins (B, E, C), pentoxifylline, heparin, and histamine [12].

In our research, it was found that patients with atypical SSNHL had a tendency to be younger (second-fourth decades), lack diabetes and hypertension, have better initial and final hearing, and rapid improvement in both subjective and objective results. Studies concerning SSNHL focused on patients with classical hearing loss, an early onset within three days, in a sensorineural pattern of at least 30 dB over three consecutive frequencies. Patients who did not meet these criteria tended to be excluded from most studies [1-5]. These patients had minimal hearing impairment, which was compatible with the complaint. Therefore, it is important to start a treatment protocol, and the follow-up schedule should be planned. In this research, the patients with control audiograms both on the fifth day and first month were included in the study to understand the effect of one bolus dose of IV systemic steroids.

Atay et al. found that the time until the start of treatment had a significant effect on hearing recovery, as well as the initial audiogram pattern. The mid-frequency hearing loss of “flat” type and/or the initiation of treatment earlier than five days from the onset of symptoms seems to have positive prognostic effects on hearing recovery. It was a retrospective study, and classical SSNHL patients were included in the study; the time until the start of treatment was analyzed as one of the variables [4]. In the design phase of this study, it was planned to include atypical SHL in the first week of the onset of the complaints. However, during the data analysis, it was noticed that the patients were given both IV steroid and betahistine protocols generally in the first five days from the initiation of the complaint. The time until the start of the treatment was in the first five days of complaints, and this may cause a higher incidence of recovery in this research compared to that in the literature.

Lee et al. studied idiopathic SHL with minimal hearing impairment and divided the patients into two groups in a cross-sectional study: group 1 with classical SSNHL and group 2 with an atypical hearing loss pattern. They found that group 2 had more rapid and more prominent hearing recovery. They pointed out that group 2 had younger patients without any systemic diseases, such as diabetes and hypertension, and these patients might be associated with acute low-tone hearing loss without vertigo (ALTHL) [3]. The definition of ALTHL is that the sum of the hearing levels at 125, 250, and 500 Hz should be greater than 70 dB, whereas the sum of the hearing levels at 2,000, 4,000, and 8,000 Hz was 60 dB or less [3,13]. Fushiki et al. studied sudden low-tone hearing loss patients and found that the ALTHL population tended to be younger than the patients with low-tone SSNHL but not in the ALTHL group [13]. In our research, the patients were of younger age (mean age: 31.5; ranging between 18 and 53 years) compared to the general age distribution of classical SSNHL, which occurs most frequently between the fourth and sixth decades. A total of 13 of the cases had high-frequency hearing impairment, whereas 27 patients had low or low-to-mid tone hearing loss, suggesting that these ALTHL patients might be included in our study.

Another possible diagnosis could be Meniere disease. Even though patients with Meniere disease and fluctuated hearing symptoms were excluded at the beginning of this study, symptoms such as hearing loss, tinnitus, and ear fullness are likely to be in Meniere disease. None of the cases in our study described dizziness, but the other complaints and audiological findings might have partially overlapped with early hydrops. Meniere disease is known to dominate in females and accompanies dizziness, different from patients in this study. Additionally, Meniere disease manifests mostly between the fourth and seventh decades of life with an increasing prevalence with age [14].

## Conclusions

SSNHL is one of the otolaryngological emergencies, which is not uncommon, and many studies have been performed to understand its pathophysiology, prognostic factors, and treatment options. However, atypical SSNHL patients have been underestimated and generally excluded from studies, and there have been a limited number of studies on this issue. These patients might be accepted as having mild hearing loss. However, when nerve injury is proven by audiograms, it is hard to decide what to do due to a lack of treatment approach. This study is important as it focuses on atypical SSNHL cases. Additionally, the present study has a few limitations, such as including a small number of patients and being retrospective. Further studies with larger group patients or prospective randomized controlled group studies are needed to define these patients and to decide how to treat this type of acute nerve dysfunction.
